# KCNQ Channels Regulate Age-Related Memory Impairment

**DOI:** 10.1371/journal.pone.0062445

**Published:** 2013-04-30

**Authors:** Sonia Cavaliere, Bilal R. Malik, James J. L. Hodge

**Affiliations:** School of Physiology and Pharmacology, University of Bristol, Bristol, Avon, United Kingdom; Imperial College London, United Kingdom

## Abstract

In humans KCNQ2/3 heteromeric channels form an M-current that acts as a brake on neuronal excitability, with mutations causing a form of epilepsy. The M-current has been shown to be a key regulator of neuronal plasticity underlying associative memory and ethanol response in mammals. Previous work has shown that many of the molecules and plasticity mechanisms underlying changes in alcohol behaviour and addiction are shared with those of memory. We show that the single KCNQ channel in *Drosophila* (dKCNQ) when mutated show decrements in associative short- and long-term memory, with KCNQ function in the mushroom body α/βneurons being required for short-term memory. Ethanol disrupts memory in wildtype flies, but not in a *KCNQ* null mutant background suggesting KCNQ maybe a direct target of ethanol, the blockade of which interferes with the plasticity machinery required for memory formation. We show that as in humans, *Drosophila* display age-related memory impairment with the *KCNQ* mutant memory defect mimicking the effect of age on memory. Expression of *KCNQ* normally decreases in aging brains and *KCNQ* overexpression in the mushroom body neurons of *KCNQ* mutants restores age-related memory impairment. Therefore KCNQ is a central plasticity molecule that regulates age dependent memory impairment.

## Introduction

KCNQ (Kv7) channels mediate a range of important physiological functions and are a hotspot of genetic diseases and therefore target for existing and novel drug treatments. In human cardiac muscle, *KCNQ1* loss of function mutations result in the most common form of cardiac arrhythmia, Long QT syndrome, while gain of functions mutations cause Short QT and atrial fibrillation [1], [2]. *KCNQ1* mutations also result in adult onset type II diabetes [3], [4]. In the nervous system KCNQ3 can heteromultimerise with either KCNQ2 or KCNQ5 subunits to form a channel that mediates a M-current, a current that is suppressed by muscarinic acetylcholine receptor activation. Because the M-current operates at resting membrane potential it is well poised to regulate membrane excitability so that when it is open it acts as a brake on action potential firing while if it is suppressed it increases neural activity and neurotransmitter release [5], [6]. These features and its broad neuronal expression allow KCNQ channels to have an important function in synaptic plasticity and memory, alcohol response and nociception [2], [7], [8]. *KCNQ2* or *KCNQ3* loss-of-function mutations result in a developmental form of epilepsy called Benign familial neonatal convulsions [2], [5]. *KCNQ4* loss-of-function mutations are a common cause of autosomal dominant deafness and age-dependent hearing impairment [9], [10]. M-current inhibitors increase excitability of cholingeric neurons and have shown some promise as cognitive enhancers in models of dementia. Conversely, M-current openers are of great interest as anticonvulsants, analgesics and treatments of psychiatric diseases [2], [5]. In mice expression of human dominant negative KCNQ2 transgene in hippocampal neurons increases neural excitability and results in associative memory deficits [7].

*Drosophila* has a single KCNQ (dKCNQ) channel that is most highly expressed in the nervous system [11]–[13], but like mammalian KCNQ1 [2] is also expressed in the heart. dKCNQ encodes a slowly activating and deactivating Kv current that can be suppressed by muscarinic acetylcholine receptor agonists and hence is an M-current [12], [14]. dKCNQ has been shown to have important age-dependent cardiac function, with hearts from young *dKCNQ* loss-of-function mutant flies showing arrhythmias similar to those seen in aged wildtype flies, whose hearts shows age dependent reduction in *dKCNQ* expression [13]. dKCNQ has many features of the M-current including conserved acute block by low concentrations of ethanol and broad neuronal expression [15]. Furthermore, targeted expression of *KCNQ-RNAi* in *Drosophila* neurons increased neural excitability, while KCNQ overexpression decreased excitability *in vivo. dKCNQ* loss-of-function mutant flies increased ethanol sensitivity and tolerance with acute activation of dopaminergic neurons by heat-activated TRP channel or *KCNQ-RNAi* expression shown to produce ethanol hyperexcitability [15]. In this study we characterise the role of *dKCNQ* mutants on memory, showing that *dKCNQ* expression decreases in the brain with aging and is linked to age-dependent cognitive deficits.

## Materials and Methods

### Drosophila Stocks

The *KCNQ* deletion mutant contained an imprecise excision of the *EP2074* element (*KCNQ^186^*) that removes all the 5′ and transmembrane regions of the channel and therefore is a null [13]. The *KCNQ* control was a precise excision of the element (*KCNQ^97^*) leaving the gene completely intact [13]. *uas-KCNQ* flies allowed *Gal4* promoter driven overexpression of *KCNQ* [13] while *uas-KCNQ-RNAi* (Bloomington stock 27252) allowed *Gal4* targeted knockdown of the channel. The *KCNQ* stocks were kind gifts of Dr Rolf Bodmer. *Elav-Gal4*, *uas-mCD8-GFP* and *OK107-Gal4* [16] were gifts from Dr Leslie Griffith. *OK107-Gal4, Gal80^ts^* [17] was a gift from Dr Yi Zhong. *c305a-Gal4* [18], *MB247-Gal4* [19] and *Amn(c316)-Gal4* [20] stocks were gifts of Dr Scott Waddell. Wildtype flies were *Canton S w-* (*CSw-*) from a stock previously maintained in the Waddell lab. All *KCNQ* mutant, *Gal4* and *uas* lines were out crossed with the relevant *CSw-* line prior to behavioural analysis. All genotypes and all other crosses were raised on corn-meal agar medium at 22±2°C and 60±10% humidity under 12∶12 hr light-dark cycle.

### Immunohistochemistry

Adult fly brains were dissected in HL3.1 (70 mM NaCl, 5 mM KCl, 10 mM NaHCO_3_, 115 mM sucrose, 4 mM MgCl_2_ 5 mM trehalose, 1.5 mM CaCl_2_, and 5 mM HEPES, pH 7.3) and isolated brains were fixed in 4% paraformaldehyde in HL3.1 for 30 min before being washed in HL3.1 [21]. The samples were permeabilised in HL3.1 with 0.1% triton X (HL3.1-Tx) for 1 hr, and then blocked for 1 hr in HL3.1-Tx with 0.1% BSA and 2% normal donkey serum (HL3.1-Tx-BSA-NDS). In order to the visualise the mushroom body and antennal lobe, brains were incubated with (1∶2000) rabbit anti-*Drosophila* DLG (PDZ1-2) a protein known to be highly expressed in these memory-related structures [22] overnight at 4°C in HL3.1-Tx-BSA-NDS. After washing three times in HL3.1-Tx for 20 min, the brains were incubated with anti-rabbit Alexa-648 conjugated secondary antibody (1∶400 in HL3.1-Tx-BSA-NDS) for 2 hr at room temperature. Finally the brains were washed three times HL3.1-Tx before being mounted in Vectorshield (Vector Laboratories). Samples were stored at 4°C in the dark until examination using a Leica TCS SP5 confocal microscope. The endogenous *KCNQ* expression pattern was determined by visualising membrane targeted GFP expressed using *KCNQ-Gal4* reporter lines (*KCNQ^NP3423^-Gal4, uas-mCD8-GFP*).

### Olfactory Aversive Conditioning

All experiments were performed at 25°C and 70% humidity under red light using the olfactory aversive conditioning protocol [23]. Groups of ∼100 1–4 day old male and female flies received either 1 cycle of training during which they were exposed sequentially to one odour (conditioned stimulus, CS+; 3-octanol (1∶74) or 4-methylcyclohexanol (1∶57) diluted in mineral oil) for 1 min paired with electric 60 V DC shock (US) and then to a second odour (CS-; the reciprocal odour) for 1 min without electric shock separated by a 30 sec rest period when they were exposed to fresh air. Memory was measured after 1 (∼2 min memory) training session at the choice point of the T-maze. To measure STM, flies were trained with 1 training cycle were stored for 1 hr and then allowed to distribute in the T-maze. LTM was assessed by giving flies either 5 cycles of spaced training cycles separated by 15 min rest intervals and then storing the flies for 24 hr before distribution in the T-maze. A performance index (PI) was calculated as the number of flies that distributed in the CS- arm minus the flies in the CS+ arm, divided by the total number of flies. Therefore a PI of 1.0 would be equivalent of 100∶0 distribution where all the flies avoided the CS+ (perfect memory), while a 50∶50 distribution would give a PI of zero (no memory). To test the effect of ethanol on *Drosophila* learning, flies were kept in bottles containing instant media (Formula 4–24 (R); Carolina biological supply company, Burlington, NC, USA) containing 10% ethanol in water containing a small amount of blue dye (0.05% Bromophenol blue) ∼12 hr before testing. The controls were given the same water-blue dye solution but lacking ethanol. The blue dye was used to monitor whether the flies had actually drunk the ethanol solution; this was confirmed as all the flies had blue abdomens prior to the test. For *OK107-Gal4, Gal80^ts^* experiments [17] flies were raised at 18°C and then shifted to 30°C allowing *KCNQ* transgene expression 1–2 days prior and during behavioural testing. Olfactory acuity was quantified by exposing naive flies to the odour versus air in the T-maze during a 2 min test trial. The performance index was calculated by counting the number of flies avoiding odour divided by total number flies. Shock reactivity was quantified by placing grids in each arm of the T-maze, and applying shock via the grid in one arm of the maze during a 2 min test trial. The performance index was calculated by counting the number of flies avoiding shock divided by total number flies [24]. Ethanol avoidance was quantified by placing a solution of 40% ethanol in the odour cup of one arm of the T-maze during a 2 min test trial. The performance index was calculated by counting the number of flies avoiding 40% ethanol divided by total number flies. All statistical analysis for behavioural data were performed and plotted with Graphpad Prism software.

### Quantitative RT-PCR

Age-matched flies were frozen in liquid nitrogen and decapitated by vortexing. Heads were collected and an equal number of heads from each genotype were homogenised. Trizol was added directly in to the homogenised heads and RNA was extracted according to manufacturer’s instructions (Invitrogen). RNA was DNAase treated (Ambion Inc) and reverse-transcribed (Retroscript, Ambion). *KCNQ* mRNA was measured using a TaqMan Kit(KCNQ - Dm01846741_g1) and was normalised to *Rpl23* (Rpl23-Dm02151827_g1) mRNAas a control allowing standardisation between the samples for aging experiments. The cDNA concentration was measured using Roche’s Light Cycler system and using multiplexing on a Stratagene Mx3000P system (Stratagene). All statistical analysis of data were performed and plotted with Graphpad Prism software.

## Results

### KCNQ Signalling Regulates Mushroom Body Dependent Associative Memory

**Figure 1:**
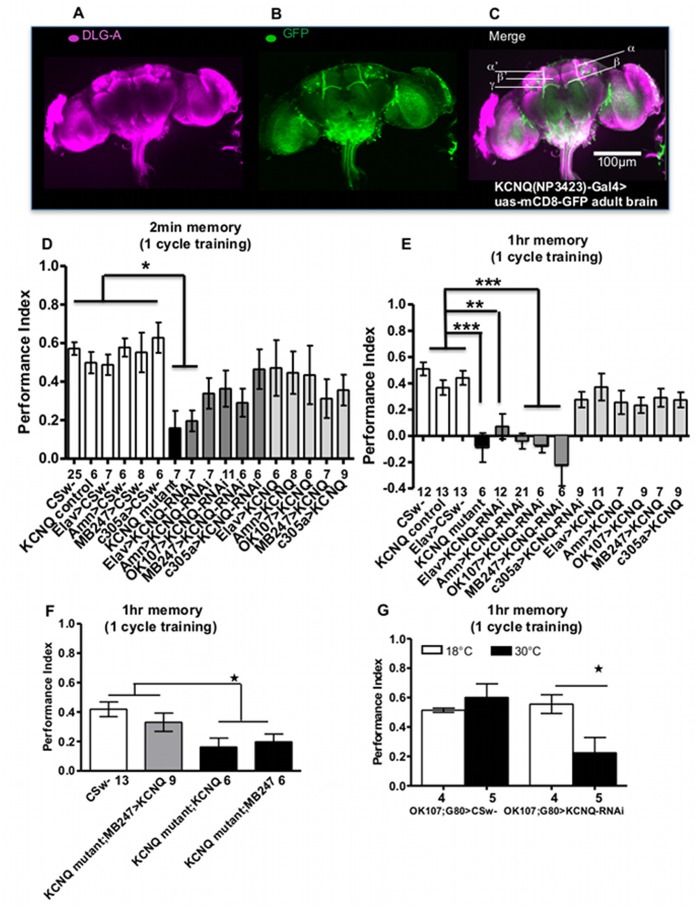
KCNQ signalling is required in the mushroom body α and β neurons for short-term memory. **A**.**–C**. Adult brains containing a Gal4 enhancer trap (KCNQ^NP3423^) in the KCNQ gene locus revealed broad neuronal expression of KCNQ (labelled by membrane bound GFP in green (**B**)) especially in the fly memory structures of the mushroom body α and β neurons and surrounding neurons known to be visualised by DLG-A (Ruiz-Cañada et al., 2002) staining (in magenta (A), co-localised structures in white (C)). **D**. Initial (2 min) memory was reduced in the KCNQ mutant (black bar) and flies with reduced KCNQ levels (dark grey bars) in all neurons (Elav-Gal4, uas-KCNQ-RNAi) (p<0.05) compared with controls (CSw-, KCNQ control, and Gal4, +, white bars) but did not lead to any change in memory (p>0.05) between the remaining genotypes. **E**. KCNQ mutants and flies with reduced KCNQ in the mushroom body (OK107-Gal4 or MB247-Gal4, uas-KCNQ-RNAi), DPM (amn-Gal4, uas-KCNQ-RNAi) (p<0.001) or all (Elav-Gal4, p<0.01) neurons have a significant reduction in 1 hr STM compared to controls (CSw-, KCNQ control and Gal4, +), while KCNQ overexpression (light grey bars) had no effect (p>0.05) with these promoters. **F**. Mushroom body α/β neuron expression of the KCNQ transgene in the KCNQ mutant background (KCNQ mutant; MB247-Gal4, uas-KCNQ) rescued the KCNQ mutant memory deficit with its memory being greater (p<0.05) than KCNQ mutant with Gal4 or uas alone (KCNQ mutant; MB247-Gal4 and KCNQ mutant; uas-KCNQ) but statistically indistinguishable (p>0.05) from control (CSw- wildtype) levels. Data in D-F were analysed by 1-way ANOVA with Bonferroni post-hoc test. **G.** 1 hr memory was measured in OK107-Gal4, Gal80^ts^, uas-KCNQ-RNAi and OK107-Gal4, Gal80^ts^, CSw- control flies raised at 18°C throughout development and then tested at 18°C conditions that prevent transgene expression (white bars). These scores were compared to the 1 hr memory of the same genotypes raised at 18°C throughout development and then shifted to 30°C allowing KCNQ transgene expression 2 days prior and during behavioural testing (black bars). 2-way ANOVA indicates significant differences due to interaction between temperature and genotype (p = 0.0195). Post-hoc analysis showed OK107-Gal4, Gal80^ts^, uas-KCNQ-RNAi had less (p<0.05) memory at 30°C compared to flies at 18°C flies (∼100 flies per n). In this and all subsequent figures, error bars represent SEM with no asterisk p>0.05, *p<0.05, **p<0.01 and ***p<0.001. n is denoted by the number between the x axis and genotype names with experiments performed on multiple different days (∼100 flies were used per n, unless otherwise stated).

Adult flies were tested for associative memory using the olfactory aversive conditioning [23]. Anatomically, neurons critical for *Drosophila* memory include those of the mushroom body that are labelled by *OK107-Gal4* [16]; these can be subdivided into α, β and γ neurons labelled by *MB247-Gal4* (which expresses strongly in α/β neurons but only weakly in γ neurons, [19]) and α’/β’ neurons labelled by *c305a-Gal4* [18]. The mushroom body-associated dorsal paired medial (DPM) neurons are also important for memory. They express the memory gene *amnesiac* (*amn*) and can be labelled by *amn(c316)-Gal4* that expresses in the DPM [20]. KCNQ is broadly expressed in the brain [12], [15]. Using a *Gal4* promoter enhancer trap within the KCNQ gene to drive GFP expression (Figure 1B), it appears *KCNQ* is expressed in the adult mushroom body α/β and surrounding neurons (Figure 1C), structures that are known to mediate memory formation and ethanol behaviour [24]–[26]. Compared with controls (Figure1D), the *KCNQ* mutant had reduced initial memory 2 min after training, as did flies with pan-neural *KCNQ* knockdown. *KCNQ* mutants and flies with pan-neural, DPM or mushroom body neuron deficient *KCNQ* completely lacked the ability to form short-term memory (STM) assessed 1 hr after training (Figure 1E).

**Figure 2:**
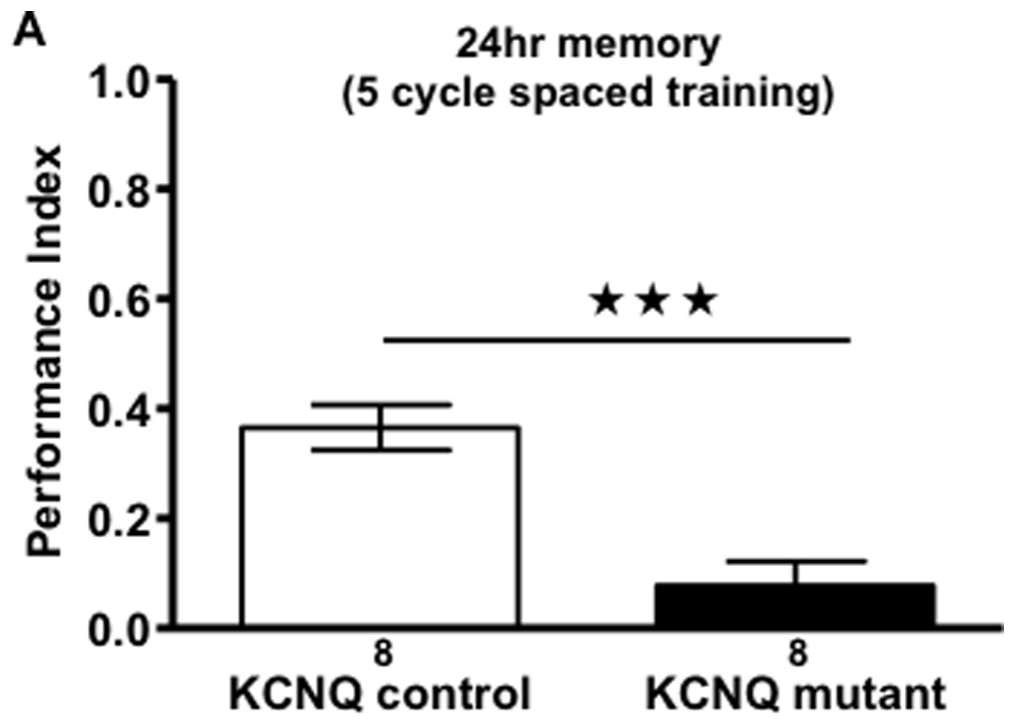
KCNQ signalling is required for long-term memory. **A**. 5 cycles of spaced training produces 24 hr LTM in KCNQ control flies that is absent (p<0.001) in the KCNQ mutants. Data were analysed with unpaired t-test.

In order to map this STM phenotype further, we selectively knocked-down *KCNQ* in different parts of the mushroom body, finding that the α/β neurons that appear to express KCNQ (Figure 1C) were required for KCNQ’s role in STM (as opposed to α’/β’ neurons (Figure 1E) which do not seem to express KCNQ (Figure 1C)). *KCNQ* overexpression in any part of the mushroom body, DPM or all neurons did not change memory measured at 2 min or 1 hr (Figure 1D–E). We also found that expression of *KCNQ* in α/β neurons using *MB247-Gal4* in a fly otherwise completely lacking *KCNQ*; rescued the *KCNQ* mutant STM defect to normal (Figure 1F). Acute reduction of *KCNQ* levels in the mushroom body was sufficient to decrease 1 hr memory compared with controls (Figure 1G), showing that KCNQ is required post developmentally to mediate physiological changes underlying memory. We then wished to determine the role of KCNQ in long-term memory (LTM) which is formed after spaced training and lasts about 7 days and is protein synthesis and CREB dependent [17], [27]. We found that the *KCNQ* mutant showed a drastic reduction in LTM (Figure 2A). No difference in ability of the flies to sense the odour (Figure S1A–B) or shock (Figure S1C) was observed between genotypes, showing that *KCNQ* mutants do not change peripheral sensory processing, but rather the memory defects are due to loss of KCNQ function in the mushroom body.

### Drosophila Display Age Dependent Memory Deficits that are Rescued by Mushroom Body KCNQ Expression

**Figure 3:**
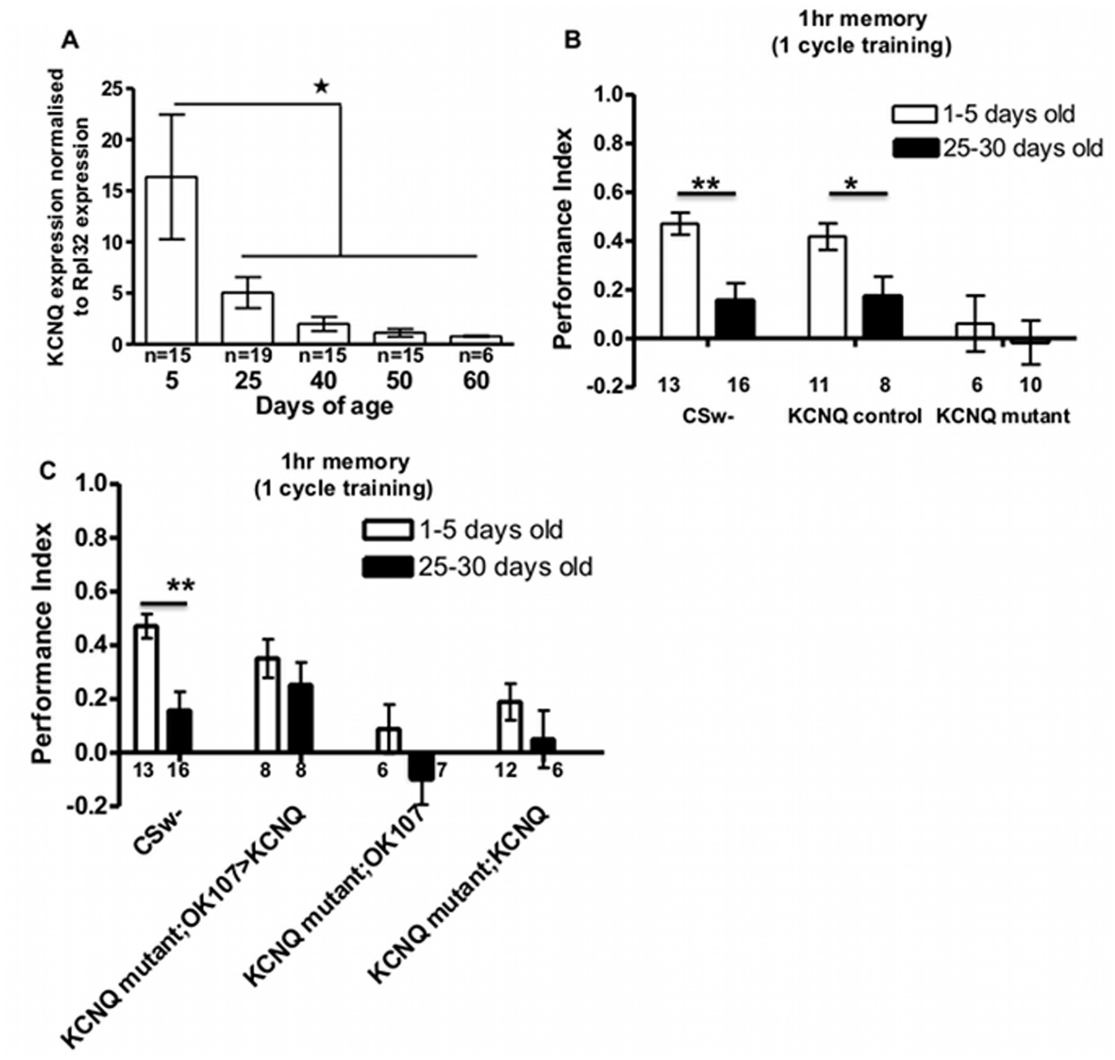
KCNQ mediates age-related memory impairment. **A**. Quantitative RT-PCR data show a dramatic age dependent reduction (p<0.05) in KCNQ expression in adult brains (20 flies per n). **B**. 1 hr memory after 1 cycle training was compared between young (1–5 days old, white bars) and aged (25–30 days, black bars) adults. 2-way ANOVA indicates significant differences in memory due to age (p = 0.0013) and genotype (p = 0.0008). Post-hoc analysis revealed that memory becomes significantly impaired in aged as opposed to young CSw- wildtype (p<0.01) and KCNQ control (p<0.05) flies. KCNQ mutant flies had equally low (p>0.05) memory whether young or old. **C**. Overexpression of KCNQ in the mushroom body rescues memory impairment of young and old KCNQ mutant flies. 2-way ANOVA indicates significant differences in memory due to age (p<0.01) and genotype (p<0.001). Post-hoc analysis revealed that memory becomes significantly impaired in aged as opposed to young CSw- wildtype (p<0.01), while the memory of KCNQ mutant; OK107-Gal4, uas-KCNQ rescue flies stays similarly high (p>0.05) in young and old flies as opposed to KCNQ mutant with Gal4 or uas alone (KCNQ mutant; OK107-Gal4orKCNQ mutant; uas-KCNQ) whose memory was similarly low in young and old flies (p>0.05).

Fly associative memory is known to decrease with age [28] and *KCNQ* expression decreases in old fly hearts leading to age related cardiac impairments [13]. Therefore we decided to test whether or not KCNQ was involved in age dependent memory decline. We first determined *KCNQ* expression in heads over the lifespan of *Drosophila* (∼50 days, [28]) using quantitative RT-PCR. We found that by 25 days *KCNQ* expression had declined to about 10% of the level of 5 day olds (Figure 3A). Therefore, we tested STM of young (1–5 day old) as opposed to aged (25–30 day old) flies. Whereas control flies displayed an age dependent decrease in 1 hr STM (Figure 3B), *KCNQ* nulls were completely unable to form STM whether they were young or old. Previous experiments have implicated the amn DPM and mushroom body neurons in mediating the effect of age on 1 hr memory [28]–[30], with expression of a *PKA* transgene in mushroom body neurons restoring age-related memory impairment. We therefore overexpressed KCNQ in the mushroom body (Figure 3C) of KCNQ mutants and demonstrate rescue of age-dependent memory impairment. These experiments are consistent with decreases in KCNQ signalling being central forage dependent decrements in memory. This experiment also confirms that in young flies expression of *KCNQ* using mushroom body *Gal4* lines (Figure 1F and 3C) in a fly otherwise completely lacking *KCNQ* rescues the *KCNQ* mutant STM defect to normal.

### Ethanol Disrupts Memory an Effect Mimicked by KCNQ Mutation

**Figure 4:**
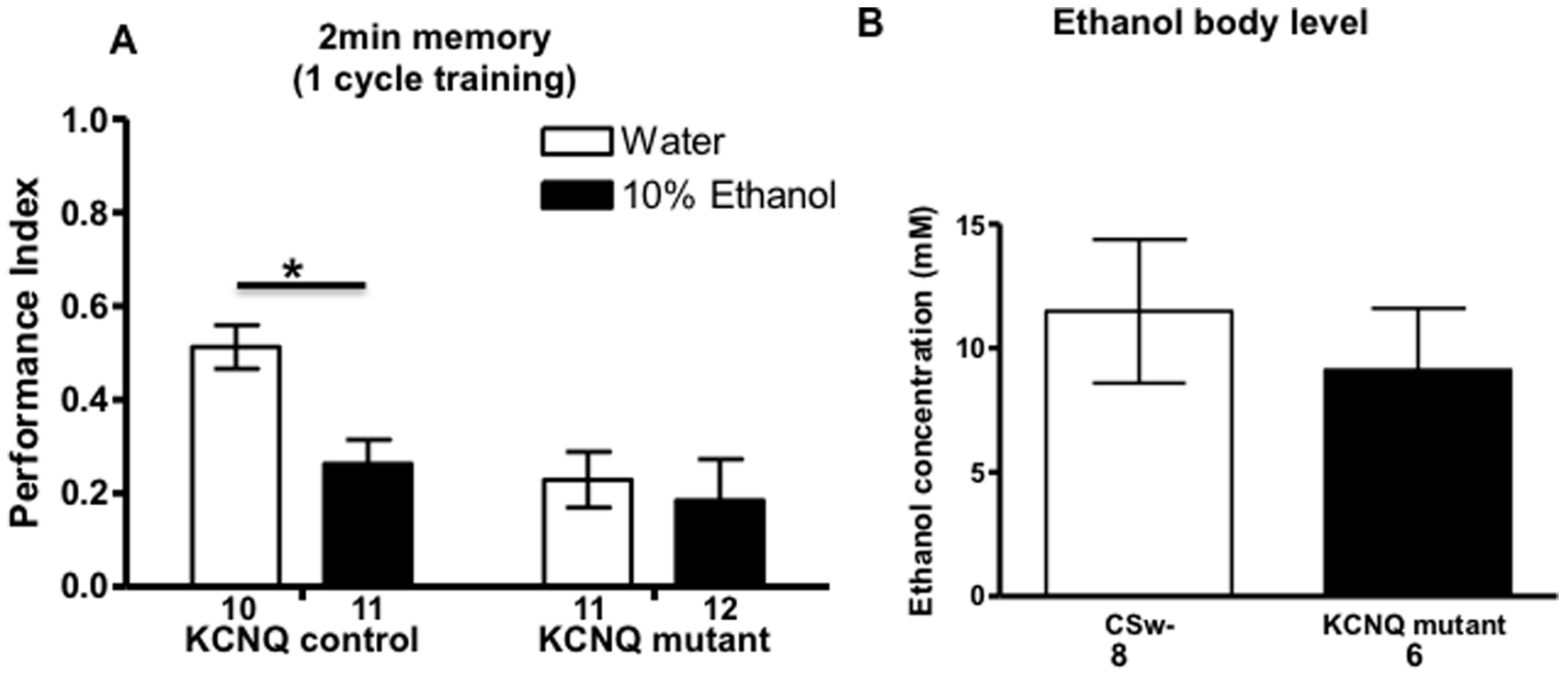
Ethanol disrupts memory in wildtype flies an effect removed by the KCNQ mutation. **A**. Flies received an overnight (∼12 hr) exposure to 10% ethanol and were tested for 2 min memory. 2-way ANOVA showed a significant effect due to genotype and ethanol (p<0.05). Post-hoc analysis showed that in KCNQ control ethanol caused a reduction (p<0.05) in memory compared to water. The reduction in memory was removed by the KCNQ mutation that had similarly low memory with or without ethanol (p>0.05). **B**. Ethanol content of KCNQ mutant and control (CSw- wildtype) flies exposed to 10% ethanol solution for ∼12 hr was similar (p>0.05, unpaired t-test, 20 flies per n).

Previous work has shown that many of the molecules and plasticity mechanisms underlying changes in ethanol behaviour and addiction are shared with those of associative memory with ethanol known to disrupt synaptic plasticity and memory in humans [29], [30]. Furthermore ethanol has been demonstrated to directly inhibit the M-current in dopaminergic neurons of the ventral tegmental area (VTA) a region of the brain important for ethanol reinforcement [26], [31]. Likewise it has been shown that dKCNQ shows conserved blockade by ethanol, with reduction in KCNQ function causing increased ethanol sensitivity and tolerance via changes in dopamine neurons [15]. Consequently we investigated whether or not ethanol disrupted fly memory. Wildtype flies were exposed to 10% ethanol solution for ∼12 hr and then immediately tested for 2 min memory (Figure 4A), although the flies did not appear sedated or intoxicated after the exposure or during the memory test, ethanol was found to reduce their memory. As KCNQ maybe a direct target of ethanol that is required for memory, we tested the *KCNQ* mutant and found that this resulted in a loss of the reduction in memory. This suggests that KCNQ is the plasticity molecule blocked by ethanol interfering with memory. No change in ethanol content was found between genotypes and was ∼10 mM at the time of the memory test (Figure 4B), this would be sufficient to block a significant proportion of neural KCNQ channels (IC_50_ = 19.8 mM, [15]).

## Discussion

*Drosophila* KCNQ displays conserved electrophysiological and pharmacological properties with mammalian neuronal KCNQ2/3 channels, with both mediating a slowly activating and non-inactivating Kv current called a M-current due to its suppression by muscarinic acetylcholine receptors [12], [14]. In the hippocampus, expression of a dominant negative human KCNQ2 transgene was found to suppress the M-current [7]. This resulted in a decrease in the afterhyperpolarization (after a train of action potentials, the increase in Ca^2+^ activates K^+^ channels leading to a pronounced hyperpolarization) and caused deficits in associative memory. Kv channels have also been implicated in plasticity underlying fly memory [32]–[35] consistent with the conspicuous expression of Kv currents in mushroom body neurons [34], [36], [37]. We have extended these studies by showing dKCNQ has a role in immediate memory, STM and LTM without peripheral defects. Targeted reduction of *KCNQ* in the mushroom body α/β neurons was sufficient to reduce memory, while expressing *KCNQ* in the same neurons in a fly otherwise completely lacking *KCNQ* rescued the *KCNQ* mutant memory phenotype to normal (Figure 1F). These results show KCNQ is required in the mushroom body α/β neurons in order for the fly to form STM. We found that the KCNQ memory phenotype resulted from reduced KCNQ function in the adult mushroom body (Figure 1G), showing that the role of KCNQ in formation is an acute physiological one as opposed to a developmental one. Mushroom body and DPM neurons have been suggested to respond to acetylcholine [27], [38]–[42] and muscarinic acetylcholine receptor activation is known to suppress the dKCNQ current [12], [14]. Therefore cholinergic stimulation would be expected to close dKCNQ channels causing depolarization and increasing firing and/or release from the DPM and mushroom body neurons. This might be expected to result in induction of plasticity and strengthening of recurrent activity in the DPM-mushroom body loop that could potentially consolidate memory. In addition the DPM neurons are thought to be serotonergic with serotonin playing an important role in the DPM mediated memory [43], interestingly serotonin has be shown inhibit KCNQ currents in mammalian neurons [44], [45], it is not known if this is also true for KCNQ currents in *Drosophila* neurons.

The effect of KCNQ mutation on LTM maybe through disruption of appropriate changes in resting membrane potential of memory neurons, which are required to remove the Mg^2+^ block of *Drosophila* NMDA glutamate receptors that is necessary for LTM and CREB-dependent gene expression [46].

On the basis of our data, high ethanol levels would be expected to cause significant or complete KCNQ blockade [15], disrupting synaptic plasticity and memory formation, thereby leading to alcohol induced amnesia or blackout. Consistent with this proposition we found that ethanol disrupts fly memory, an effect that was removed in the *KCNQ* mutant background (Figure 4A). This suggests that KCNQ is a key molecule that ethanol interacts with in the plasticity machinery involved in memory. Given the conserved role of mammalian KCNQ in ethanol response and memory [15], we suggest that it is likely this will also be the case for mammals.

Fly memory is known to reduce with age [28], as does *KCNQ* expression and function in the heart [13]. We found that *KCNQ* brain expression dramatically decreases with age and this is accompanied by an age-dependent decrease in memory (Figure 3A). Young *KCNQ* mutant flies that have low levels of *KCNQ* comparable to the low levels of *KCNQ* in aged wildtype flies have comparably reduced levels of memory. As mushroom body neurons are known to be important for mediating changes in memory performance with age [28], [29] and mushroom body knockdown of *KCNQ* completely removed 1 hr memory (Figure 1E), we overexpressed a *KCNQ* transgene in the mushroom body (Figure 3C) of a fly otherwise completely lacking *KCNQ* and found that this restored age-related memory impairment with young and old flies having similarly high memory. In summary we show reduction in KCNQ function in mushroom body neurons mediates age-dependent cognitive decline.

In mammals, KCNQ specific modulators have been suggested to alleviate memory deficits associated with age related memory diseases such as Alzheimer’s disease [2]. It is not clear how KCNQ-mediated mechanisms may affect memory in aged animals. However, based on the contribution of KCNQ2/3 to hippocampal afterhyperpolarizations and memory [7], one candidate mechanism would involve reduced neuronal KCNQ, as this modulates afterhyperpolarization duration that is known to change with memory and in aged animals [47]–[49]. Recently, KCNQ channels have been implicated in age-dependent decrements in the memory of primates [50], suggesting that KCNQ function in cognitive impairments accompanying aging are likely conserved from flies to humans. Furthermore, PKA signalling has been implicated in age-related memory impairment in flies and mammals [28], [29], [50], [51] with the mammalian KCNQ channel open state being increased by PKA [50], [52], suggesting that the mushroom body neuron KCNQ-mediated memory and age-dependent memory defects maybe due to an interaction with PKA.

The genetic and experimental tractability of *Drosophila* combined with its ∼50 day lifespan and molecular conservation with human make it a convenient and powerful genetic model [28] to study further the age-dependent KCNQ cognitive deficits. We have shown that KCNQ neuronal function in memory and ethanol response are evolutionarily conserved with mammals, allowing further development of *Drosophila* models of KCNQ neuronal function and channelopathies to elucidate KCNQ signalling networks, mechanisms of aging and potential screening for new disease therapies.

## Supporting Information

Figure S1 A. KCNQ channel mutants display normal olfactory acuity and shock reactivity. Experimental and control (*CSw-* wildtype or *KCNQ* control) flies similarly (p>0.05) avoided OCT **A**. and MCH **B**. odour used in the memory assay. **C**. Experimental and control (*CSw-* wildtype or *KCNQ* control) flies similarly (p>0.05) avoided the arm of the T-maze delivering 60 V DC electric shock. Data in A-C were analysed by 1-way ANOVA with Bonferroni post-hoc test.
(TIFF)
